# The tRNA methylation activity and specificity of NSUN2 are independent of Archease

**DOI:** 10.1038/s41598-026-63726-2

**Published:** 2026-07-29

**Authors:** Valentina Stuchlik, Maria Waldl, Anming Huang, Moritz Kleinwächter, Anna Rocchegiani, Fatinah El-Isa, Ivo L. Hofacker, Javier Martinez, Alexandra Lusser, Elisa Vilardo

**Affiliations:** 1 Center for Anatomy & Cell Biology, Medical University of Vienna, Vienna, Austria; 2 Department of Theoretical Chemistry, University of Vienna, Vienna, Austria; 3 Vienna Doctoral School in Chemistry (DoSChem), University of Vienna, Vienna, Austria; 4 Institute of Computer Science and Interdisciplinary Center for Bioinformatics, Leipzig University, Leipzig, Germany; 5 Institute of Molecular Biology, Biocenter, Medical University of Innsbruck, Innsbruck, Austria; 6 Max Perutz Labs, Medical University of Vienna, Vienna Biocenter Campus (VBC), Vienna, Austria; 7 Doctoral School, Vienna Biocenter PhD Program, a Doctoral School of the University of Vienna and the Medical University of Vienna, Vienna, Austria; 8 Faculty of Computer Science, Research Group Bioinformatics and Computational Biology, University of Vienna, Vienna, Austria

**Keywords:** Biochemistry, Cell biology, Developmental biology, Evolution, Molecular biology

## Abstract

Transfer RNAs (tRNAs) undergo extensive maturation, including modifications essential for translation efficiency and fidelity, as well as for structural stability. The modification cytosine-5 methylation (m^5^C) is catalyzed by NSUN2 at multiple positions within distinct structural regions of a subset of tRNAs, yet the determinants of its substrate specificity remain elusive. In archaea, the protein Archease has been implicated in modulating the cytosine specificity of the NSUN2 ortholog Trm4, whereas mammalian Archease was shown to be involved in pre-tRNA splicing. Here, we investigated whether mammalian Archease influences NSUN2 in a similar way like the archaeal enzyme. Using short hairpin RNA-mediated depletion of Archease in mouse embryonic stem cells and in vitro methylation assays with recombinant proteins, we assessed both the catalytic activity and substrate selectivity of NSUN2 in the presence or the absence of Archease. Our results indicate that neither the efficiency nor positional specificity of NSUN2-mediated tRNA m^5^C methylation is affected by Archease. These findings indicate that, unlike in archaea, Archease does not regulate tRNA methylation by NSUN2 in mammals, highlighting an evolutionary divergence in the functional role of Archease in tRNA maturation pathways.

**Supplementary Information:**

The online version contains supplementary material available at https://doi.org/10.1038/s41598-026-63726-2.

## Introduction

Transfer RNAs (tRNAs) in all domains of life are initially transcribed as precursor molecules, and reach their mature, functional form after extensive maturation involving nucleotide addition and removal, and chemical modification^1^. The diverse maturation steps are catalysed by a plethora of enzymatic activities, many of which are constituted by multi-subunit enzymes^2,3^. Despite accumulating knowledge about the architecture of modification enzymes^4^, the determinants of their substrate selection and specificity, and the specific role of non-catalytic subunits remain poorly understood. A few modification enzymes are able to install modifications at different sites within a tRNA, in diverse sequences and structural contexts. One such case is the cytosine-5 methyltransferase NSUN2, which is responsible for the methylation of tRNAs at positions 34, 40, 48, 49 and 50 (according to canonical tRNA positions numbering^5^, but only in a subset of tRNAs^6–8^(Supplementary Fig. 1a). How NSUN2 selects its position specificity, ranging from a single stranded region in the anticodon loop, to the double-stranded T-stem, remains unclear.

Work in the hyperthermophile archaeon *Pyrococcus abyssi* indicateded that the protein Archease affects the specificity of the cytosine-5 methyltransferase Trm4, which is the archaeal ortholog of mammalian NSUN2^9^. Specifically, Archease from *P. abyssi* restricted the introduction of m^5^C catalyzed by Trm4 to C49 of tRNA^Asp^, in particular under high temperature. More recently, it was shown that human Archease is a component of the pre-tRNA splicing machinery, promoting efficient ligation of tRNA halves by the tRNA ligase complex^10^.

Here, we investigated whether the regulatory role of Archease on Trm4 is conserved in mammals. Using both short-hairpin RNA-mediated knockdown (KD) of Archease in mouse embryonic stem cells (mESCs), and recombinant human Archease in tRNA methylation assays, we show that the tRNA m^5^C methyltransferase activity and tRNA substrate specificity of NSUN2 in vivo and in vitro is not influenced by Archease, pointing to a divergence of the role of Archease in tRNA maturation in different domains of life.

## Materials and methods

### Cell culture and archease knock down

mESCs (KH2) were cultured in ESC LIF+2i medium, consisting of DMEM high glucose with GlutaMAX (Gibco), 15% embryonic stem cell-qualified fetal bovine serum (Gibco), 1× non-essential amino acid mix (Gibco), 1 mM sodium pyruvate (Gibco), 0.05 mM β-mercaptoethanol (Gibco), 10 µg/mL leukemia inhibitory factor (LIF, Sigma), 3 µM CHIR99021, and 1 µM PD0325901 (Axon Medchem). Cells were maintained on gelatin-coated tissue culture dishes at 37 °C in a humidified atmosphere with 5% CO₂.

To generate mESCs with stable knockdown of *archease (Zbtb80s)*, two complementary DNA oligonucleotides encoding a short hairpin RNA (shRNA) targeting *archease* (Supplementary Table 1) were hybridized and cloned into the *Age*I/*Eco*RI sites of the pLKO.1-puro vector (Addgene #8453). Lentiviral particles were produced in HEK293T cells by co-transfecting the recombinant pLKO.1 vector with the psPAX2 packaging plasmid and the pMD2.G envelope plasmid, following the Addgene pLKO.1 protocol (https://www.addgene.org/protocols/plko/). To concentrate the lentiviral particles, 8.5% polyethylene glycol (PEG) 6000 and 0.4 M NaCl were added to the viral supernatant, followed by overnight incubation at 4 °C. The mixture was then centrifuged at 7,000 g for 30 min at 4 °C. The resulting virus pellet was resuspended in 200 µL of nuclease-free water. For transduction, mESCs were seeded in ESC LIF+2i medium and cultured overnight. The following day, 1 µg/mL polybrene and 5 µL of the concentrated virus particles were added to the cells. After 24 h of incubation, the medium was replaced with fresh ESC LIF+2i medium, and the cells were cultured for an additional 24 h. Puromycin was then added to a final concentration of 5 µg/mL to select for stably transduced cells. After seven days of selection, cells were maintained in 2 µg/mL puromycin.

Mouse embryonic fibroblasts (MEFs) KO for *Nsun2*^*7*^ were a generous gift of Matthias Schaefer (Medical University of Vienna). MEFs were cultivated in DMEM high glucose (Gibco) supplemented with 10% fetal bovine serum, 2 mM L-glutamine, and 1x non-essential amino acids (Gibco).

### Reverse transcription (RT) qPCR

Total RNA of Archease KD and control cells was isolated using Trizol (Sigma-Aldrich), followed by DNase I treatment (1 U/µg RNA; NEB) for 30 min at 37 °C and purification by acid phenol/chloroform extraction (Roth) and ethanol precipitation. Reverse transcription was performed using the GoScript Reverse Transcription System (Promega) with random primers (Promega) according to the manufacturer’s instructions. qPCR was conducted using 2× Luna Universal qPCR Master Mix (NEB), 0.5 mM gene-specific primers (Supplementary Table 1), and 40 ng of cDNA on a QuantStudio 3 Real-Time PCR System (Thermo Fisher Scientific). *Archease* mRNA expression was calculated relative to *TATA binding protein* (*TBP*) transcripts using the QuantStudio 3 Comparative Ct program for SYBR Green-based experiments.

### Western blotting

mESCs were lysed in 200 µL of 0.5× Laemmli sample buffer (62.5 mM Tris, pH 6.8, 2% SDS, 10% glycerol, 5% 2-mercaptoethanol), sonicated for 10 min in a Bioruptor Sonicator (Diagenode) at 4 °C, and centrifuged at 16,000 g for 20 min. The supernatant was collected, and an aliquot was loaded onto a 12% SDS-polyacrylamide gel for electrophoresis. Proteins were transferred to a nitrocellulose membrane using the Bio-Rad semi-wet blotting system. Membranes were blocked with 5% bovine serum albumin (BSA) in phosphate-buffered saline (PBS) and incubated overnight at 4 °C with primary antibodies (α-archease^11^, 1:500; mouse α-tubulin, 1:10000, Thermo Fisher MA5-16308). After washing with PBS, membranes were incubated with Alexa Fluor™ Plus 488-conjugated secondary antibody (1:5000, Thermo Fisher A32723). Following additional PBS washes, the signal was detected using a Typhoon FLA 9500 imaging system (Cytiva).

### Expression and purification of recombinant proteins

The coding sequence of human NSUN2 was amplified from the plasmid pDEST12.2-NSUN2-Flag^12^(generous gift of Tsutomu Suzuki of the University of Tokyo) with the primers listed in Supplementary Tables 1 and cloned in the sites *Nde*I and *Xho*I of pET28b(+), generating an expression cassette including a 6xHis-Tag and thrombin site at the N-terminus of NSUN2. NSUN2 was expressed in *E. coli* BL21(DE3) upon induction with 1 mM IPTG in LB medium at 18 °C over-night. Bacteria were lysed by sonication in buffer A (150 mM NaCl, 10 mM Tris·Cl pH 7.5, 10% glycerol, 1 mM DTT, 0.2% Tween, 0.2% Protease Inhibitor Cocktail (Sigma-Aldrich)) supplemented with 0.16 mg/ml Lysozyme, 0.008 mg/ml Dnase I, and 12.5 mM imidazole; the lysate was loaded on a Cytiva His-SpinTrap column, incubated at 4 °C for 15 min rotating and centrifuged at 100xg for 30 s at 4 °C. The column was washed once with buffer A supplemented 12.5 mM imidazole, once with buffer A, trice with buffer A supplemented with 1 M NaCl, then twice with buffer A. NSUN2 was eluted with buffer A supplemented with 300 mM imidazole. The protein concentration was estimated relative to BSA standards on an 8% SDS polyacrylamide gel followed by Coomassie staining and densitometric analysis with ImageQuant TL (Cytiva).

Human Archease was expressed and purified as described previously^13^. Activity of the purified protein was verified by performing an in vitro guanylation assay: 20 pmol recombinant human Archease were incubated with 20 pmol recombinant human RTCB in reaction buffer (10 mM Tris-HCl pH 7.5, 100 mM KOAc, 6 mM Mg(OAc)_2,_ 150 µM Spermin-HCl pH 7.5, 0.5 mM DTT, 0.5 µM ATP, 0.5% Triton-X 100 v/v, 15% Glycerol v/v and 92.5 kBq [α-^32^P]guanosine-5′-triphosphate) at 37 °C for 30 min. The reaction was stopped by adding 5x SDS loading dye (62.5 mM Tris-HCl pH 6.8, 25 mM EDTA pH 8.0,5% SDS w/v, 5% β-mercaptoethanol v/v, 0.025% bromophenol blue w/v, 50% glycerol v/v) to a final concentration of 1x and subsequent incubation at 95 °C for 3 min. The guanylation state of RTCB in the sample was determined by SDS-PAGE and autoradiography.

### In vitro methylation of *Nsun2* KO MEFs tRNA

RNA of MEFs KO for *Nsun2* was extracted with Ribozol (VWR) according to manufacturer’s instructions, and separated on a 10% polyacrylamide gel; the tRNA fraction was isolated by band excision and elution.

The tRNA fraction isolated from *Nsun2* knock-out MEFs was incubated in methylation reactions of 80 µl containing 0.15 µM recombinant NSUN2, 4 ng/µl MEF tRNA, and 25 µM S-adenosylmethionine (SAM) in reaction buffer (50 mM Tris-HCl pH 7.5, 50 mM KCl, 5 mM MgCl_2_, 0.5 µM ATP, 0.5 µM GTP, 0.5% Triton X-100, 2 mM DTT, 20 µg/ml BSA, 15% Glycerol, 0.5 units/µl Ribolock RNase inhibitor (Thermo)), with or without 0.5 µM recombinant Archease. Aliquots of 25 µl were withdrawn after 1, 10, or 120 min of incubation at either 37–40 °C. A mock control sample lacking NSUN2 and Archease was included.

Subsequently, the tRNA in the samples was phenol/chloroform extracted, precipitated, and redissolved in RNase-free water before proceeding to bisulfite conversion with the EZ RNA Methylation Kit (Zymo Research) according to manufacturer’s instructions.

### Library preparation and next generation sequencing

Total RNA was separated on a 10% polyacrylamide gel; the tRNA fraction was isolated by band excision and elution, and further processed according to the tRAM-seq protocol^14^ with the following adjustments. For the sequencing of MEF tRNA: after being, in order, deacylated, dephosphorylated and PCI extracted, the tRNA samples were ligated to a pre-adenylated adapter containing a 5 nt-long barcode (Supplementary Table 1). The libraries were then pooled for subsequent library preparation steps. The different libraries were demultiplexed during data analysis according to the barcodes. For the NGS data analysis of all experiments, the reads were mapped to individual isodecoders. For visualization, data were pooled per tRNA anticodon family. For additional details see supplementary methods in Supplementary Info File 1.

### Preparation of tRNA^Asp^ substrate

The tRNA^Asp^ substrate was generated by ligation of an in vitro transcribed RNA corresponding to nucleotides 1–48 and a synthetic RNA corresponding to nucleotides 49–76. A plasmid encoding human tRNA^Asp-GTC-2^ (nucleotides 9–76) previously described^15^ was modified to insert nucleotides 1–8 by cleaving with restriction enzymes *Eco*RI and *Bsa*I (NEB) and inserting the annealed oligoes listed in Supplementary Table 1; subsequently, the nucleotides 49–76 were removed by cleavage with *Bsa*I and *Ava*II and insertion of the corresponding annealed oligoes (Supplementary Table 1). The resulting expression cassette consisted of T7 promoter, hammerhead ribozyme, nucleotides 1–48 of the tRNA, and a 3’ HDV ribozyme. The obtained plasmid was linearized by cleavage with *Hind*III and used as template for in vitro transcription as previously described^16^. The tRNA fragment 1–48 was 3’-end repaired by incubation with T4 polynucleotide kinase (NEB), then ligated by two-way ligation to a synthetic RNA oligonucleotide corresponding to the fragment 49–76 (Supplementary Table 1) ^32^P labelled at the 5’, similar to previously described^16^.

### NSUN2 in vitro tRNA^Asp^ methylation assay

The kinetics of methylation of human NSUN2 on tRNA^Asp^ C48 and C49 was assayed by in vitro methylation. The two-way ligated tRNA^Asp^ substrate (< 10 nM) was incubated at 37 °C with 75 nM recombinant NSUN2, and 25 µM SAM in reaction buffer (50 mM Tris-HCl pH 7.5, 50 mM KCl, 5 mM MgCl_2_, 0.5 µM ATP, 0.5 µM GTP, 0.5% Triton X-100, 2 mM DTT, 20 µg/ml BSA, 15% Glycerol, 0.5 units/µl Ribolock RNase inhibitor (Thermo)), with or without 250 nM recombinant Archease. A mock control sample lacking NSUN2 and Archease was included. Aliquots of 5 µl were withdrawn at specific time points, and stopped by addition of Guanidinium-HCl (final 83 µM). For each sample, 1 µl was loaded on a 10% denaturing polyacrylamide gel followed by autoradiography to assess quality/degradation; 2 µl were digested with 0.5 U of nuclease P1 (Sigma); 2 µl were digested with 0.2 U of nuclease T2 (MoBiTec). 5‘ and 3‘ nucleoside monophosphate hydrolysates resulting from P1 and T2 digestion, respectively, were separated by thin layer chromatography on cellulose coated plates (Machery-Nagel POLYGRAM CEL 300) with buffer B as previously described^16,17^.

## Results

### Depletion of Archease does not affect the steady state levels of tRNA methylation in mouse embryonic stem cells

**Fig. 1:**
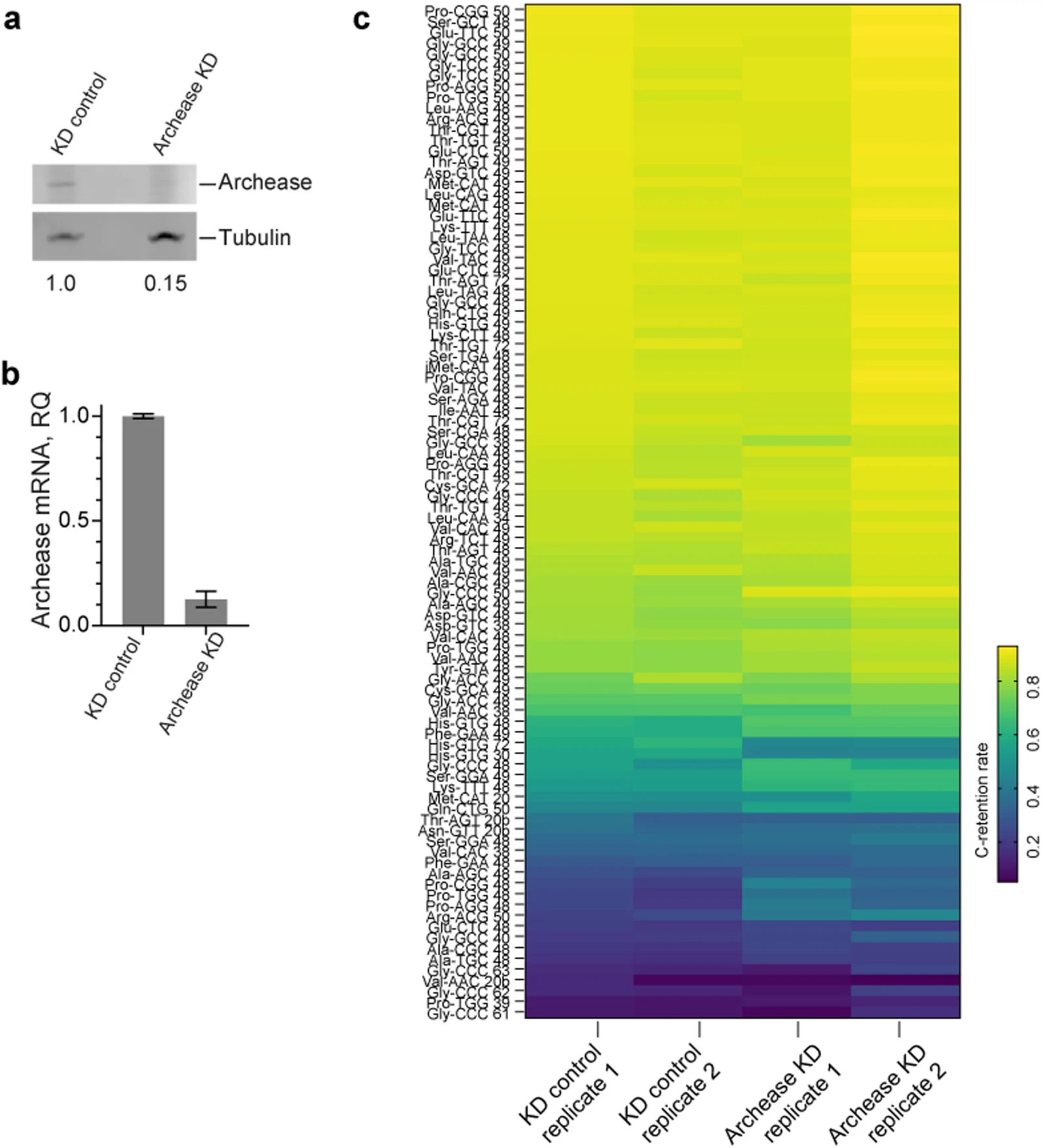
Knock down of Archease in mESCs. (**a**) The abundance of Archease protein was assayed by western blot in mESCs stably expressing a shRNA targeting Archease or a scrambled sequence (KD control). Tubulin was used as internal calibrator; calculated Archease abundance is indicated below the blot, as normalized to KD control. Shown is a representative Western blot result. Source images are available in Supplementary Figure S2a, b. (**b**) Archease mRNA levels were assayed by RT-qPCR; the y axis indicates relative quantity (RQ), normalized to KD control. Shown are mean and SD of three technical replicates (replicate amplification of same biological sample). (**c**) C-retention fraction throughout the tRNA transcriptome, as obtained from tRAM-seq analysis of bisulfite converted RNA. Data are shown per anticodon family. Only cytosines with minimum coverage of 100 reads and C-retention rate of at least 0.1 in at least one sample are shown. C-retention fractions were obtained from independent library preparation and sequencing of two independently cultivated and harvested biological samples per condition.

In *Pyrococcus abyssi*, Trm4 was reported to introduce methyl groups at multiple positions in tRNA^Asp^, spanning positions 35 to 51, whereas the addition of Archease restricted the activity of Trm4 to C49^9^. To test whether this functional interaction is conserved in mammals, we generated mESCs stably expressing an shRNA against Archease, or a scrambled shRNA control. We then processed RNA extracted from Archease KD and control mESCs for tRAM-seq, a library preparation protocol and computational analysis pipeline that we previously established for tRNA expression and modification analysis^14^, and compiled the tRNA m^5^C modification landscape of mESCs based on C-retention in NGS data after bisulfite conversion of tRNAs (Supplementary Fig. 1b). In Archease KD mESCs, we achieved a reduction in Archease levels down to about 15% compared to control cells, at both protein and mRNA level (Fig. 1a-b and S2). When comparing the extent of C-retention, which indicates m^5^C methylation, we did not observe any significant difference between tRNAs from Archease KD and control mESCs (Fig. 1c and Supplementary Info File 2). In particular, we did not observe any significant increase in C-retention at unexpected sites, suggesting that the KD of Archease did not elicit an off-target methylation activity of NSUN2 on cytosines in tRNA^Asp^ or any other tRNA.

### NSUN2 methylates tRNAs in vitro independently of Archease

**Fig. 2:**
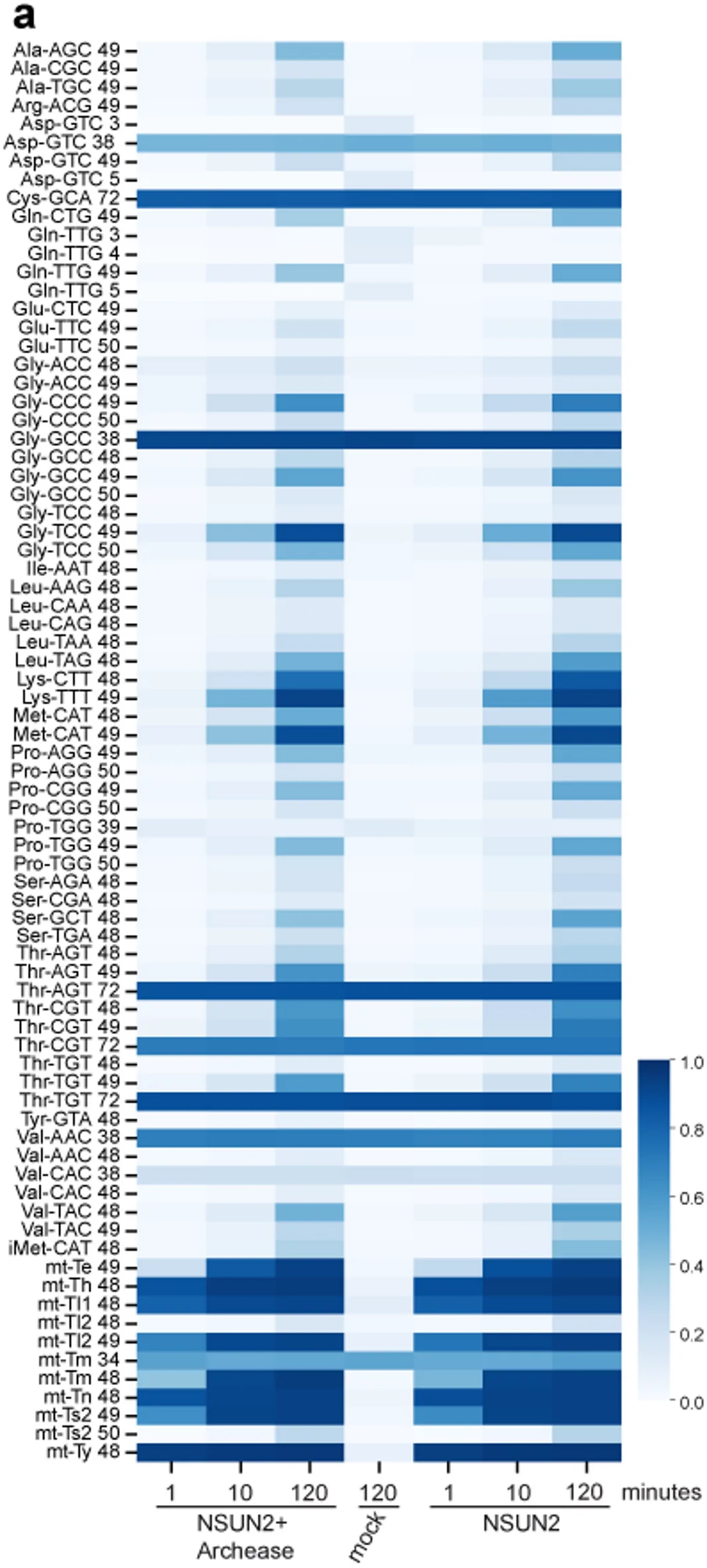
Methylation of cellular tRNAs by NSUN2 in presence or absence of Archease at 37 °C. RNA was extracted from Nsun2 KO MEFs and incubated at 37 °C with NSUN2 with or without Archease, then processed for tRAM-seq analysis. Quantification of C-retention fraction (thus m^5^C methylation) throughout the tRNA transcriptome is shown after different incubation times. Data are shown per anticodon family; on the Y axis tRNA anticodon identity and cytosine position are specified. Only positions with minimum coverage of 500 reads, where references encoding a cytosine represent a fraction of at least 0.1 of the total, and with a minimum C-retention fraction of 0.1 are shown. Sites with C-retention signal deriving from other modifications were omitted. Cytosines with high C-retention in all samples represent targets of Dnmt2 (C38), Nsun3 (C34), and Nsun6 (C72). Cytosines with C-retention higher in the mock sample than in the treated samples represent *bona fide* background due to incomplete bisulfite conversion.

Since the KD of Archease was not complete, we could not exclude that the residual Archease present may have been sufficient to regulate the specificity of Nsun2 in Archease KD cells. To test in a more controlled setting whether the presence of Archease affects the activity of NSUN2, we set up an in vitro methylation assay using recombinantly expressed and purified human NSUN2 and Archease. As substrate, we used total tRNAs extracted from Nsun2 null MEFs, whose sequences are virtually identical to human tRNAs and are devoid of any m^5^C modification at Nsun2 target sites^7^. The total tRNA was incubated in vitro at 37 °C with either NSUN2 alone or NSUN2 plus Archease, or without any recombinant protein (mock reaction). Methylation reactions were stopped at different time points and processed for analysis by tRAM-seq. Also in this case, we did not observe any substantial difference between tRNA methylated by NSUN2 alone or with the addition of Archease, with regard to the extent of C-retention (thus methylation) at known NSUN2 target sites, or at non-target cytosines (Fig. 2, and Supplementary Info File 3). In *P. abyssi*, Archease was reported to enhance the specificity of Trm4 leading to increased methylation at C49 in tRNA^Asp^ and decreased methylation at other sites in the same tRNA^9^. In our experiments, the methylation of tRNA^Asp^ C49 only reached about 30% after 2 h of incubation, and was comparable in the presence of NSUN2 alone or NSUN2 supplemented with Archease (Fig. 2). The only other C-retention site that we observed in tRNA^Asp^ was at the canonical position C38, which is known to be methylated by DNMT2/TRDMT1^18^, and indeed it was also present in the mock reaction, confirming that this position is not methylated by Nsun2, but by Dnmt2/Trdmt1 present in Nsun2 null MEFs.

In the original study reporting the effect of Archease on Trm4 specificity in the hyperthermophile *P. abyssi*, an organism that thrives at around 100 °C, Trm4 was shown to display specificity for C49 of tRNA^Asp^ at a lower reaction temperature (50 °C), while higher temperatures (80 °C) exacerbated the methylation at alternative sites^9^. Since we studied the human orthologues of the two enzymes at a standard temperature of 37 °C, we repeated the in vitro methylation assay at 40 °C, which is the highest physiologically relevant temperature for mammalian enzymes, corresponding to a febrile condition. Also at this temperature, we did not observe any difference in site specificity in tRNA methylation by NSUN2 alone or NSUN2 supplemented with Archease (Supplementary Fig. S3, Supplementary Info File 3). However, at 40 °C we observed a lower extent of tRNA methylation, indicating temperature-dependent detrimental effects on human NSUN2 activity.

### Archease does not affect the cytosine selection and methylation rate of NSUN2 on tRNA^Asp^

**Fig. 3:**
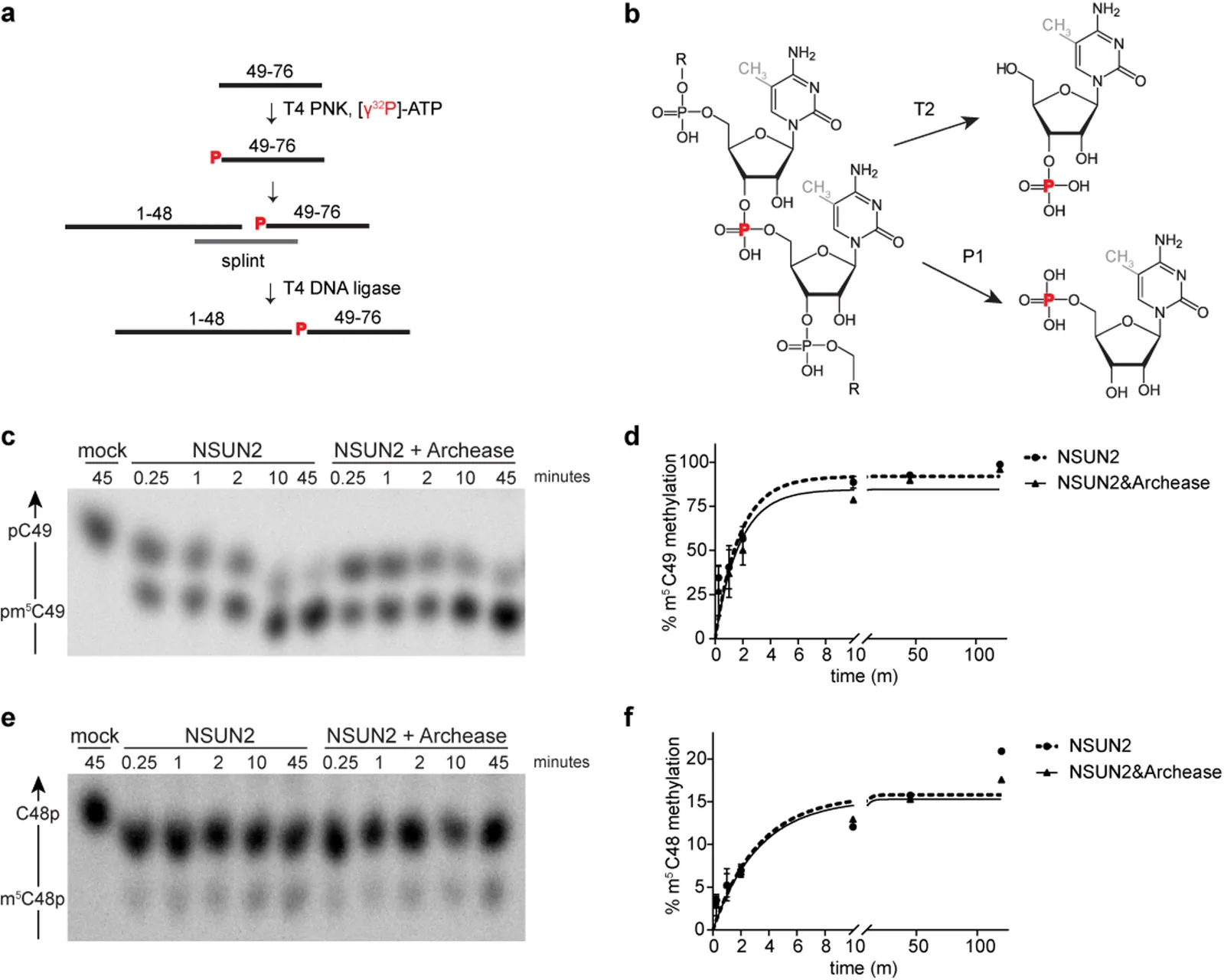
In vitro methylation of tRNA^Asp^. (**a**) A synthetic RNA corresponding to positions 49–76 of tRNA^Asp^ was labelled at the 5’ with ^32^P-ATP (indicated by red color), then ligated by splint-ligation to an in vitro transcribed RNA corresponding to positions 1–48 of tRNA^Asp^. (**b**) After incubation in vitro with NSUN2 alone or with the addition of Archease, the tRNA^Asp^ substrate radioactively labelled at the phosphate between C48 and C49 was digested either by T2 or by P1 nuclease, to generate 3’ or 5’ nucleoside monophosphates, respectively. (**c**) 5’ monophosphate hydrolysates were separated by thin layer chromatography and visualized by autoradiography to quantify methylation at C49. The arrow indicates the direction of migration of the solvent, and the identity of the spots is shown on the left. Only the relevant part of the TLC is shown; the full-length TLC is shown in Supplementary Fig. 5a (**d**) Quantification of methylation data for C49 of two independent experiments were plotted as means and SEM, and fit by nonlinear regression. (**e**) Similar to (c), 3’ monophosphate hydrolysates were separated by thin layer chromatography and visualized by autoradiography to quantify methylation at C48. Only the relevant part of the TLC is shown; the full-length TLC is shown in Supplementary Fig. 5b (**f**) Quantification of methylation data for C48 of two independent experiments were plotted as means and SEM, and fit by nonlinear regression.

Human tRNA^Asp^ is methylated by NSUN2 at canonical positions C48 and C49^2^. However, under our in vitro assay conditions, recombinant NSUN2 caused C-retention (thus m^5^C methylation) clearly detectable at C49, but barely so at C48, which is also an NSUN2 target cytosine^7^, regardless of the presence of Archease (Supplementary Fig. S4). To assay the activity of NSUN2 on tRNA^Asp^ in a more sensitive and site-specific way, we devised an in vitro methylation assay using an in vitro transcribed human tRNA^Asp^ substrate devoid of any modification and radioactively labelled at the phosphate backbone between positions C48 and C49 (Fig. 3a). This method relies on the chromatographic separation of the methylated cytosines from standard nucleoside residues, is site-specific, and allows the sampling and analysis in parallel of multiple time points in a quantitative fashion^17^. After incubation with recombinant NSUN2 with or without addition of Archease, the RNA was split in two aliquots: one was digested with nuclease P1 to generate 5‘ nucleoside monophosphates, and the other one with nuclease T2 to generate 3‘ nucleoside monophosphates, thus retaining the radioactive phosphate on the C49 or C48 residue, respectively (Fig. 3b). After thin-layer chromatographic separation, the extent of m^5^C methylation at both positions was quantified (Fig. 3c-f and Supplementary Fig. 5a-b). Under these conditions, NSUN2 was more efficient in methylating C49 compared to the analysis using MEF tRNA as substrate (Fig. 2), as near completeness was achieved in under 10 min (Fig. 3c, d); the methylation of C48 appeared to proceed more slowly, not exceeding 20% at the longest incubation time point tested (Fig. 3e, f). In either case, the presence of Archease did not affect the rate of methylation, nor the maximum methylation achieved, again showing that Archease does not affect directly the methylation activity, nor the cytosine selection by human NSUN2.

## Discussion

The recruitment of accessory subunits to tRNA maturation enzymes appears to be more prevalent in eukaryotes as compared to archaea and bacteria^19^. Some protein components of tRNA modification enzymes are associated with several modification pathways, such as in the case of the non-catalytic subunit TRMT112, which acts as partner protein for numerous methyltransferases^20^. In a few examples, non-catalytic subunits have been shown to be essential for efficient and accurate tRNA processing and modification, like in the case of the multifunctional complex of human mitochondrial RNase P^16,21^. The metabolic cost of correct maturation of tRNAs is remarkable, as the enzymes involved in tRNA modification can represent 1 to 10% of the protein-coding genes of a given genome, exceeding the number of tRNA genes themselves^22,23^. Thus, it is not surprising that higher complexity has been achieved during evolution in part by “recycling” factors across different modification activities, or even from unrelated pathways. Given the earlier reported involvement of Archease as regulator of tRNA m^5^C methylation fidelity by Trm4 in Archaea^9^, and the fact that human Archease is crucial for proficient pre-tRNA splicing^10^, it was intriguing to postulate that Archease may serve both roles in eukaryotes, possibly also mediating a feedback/coordination between two important steps of tRNA maturation: splicing and modification. However, none of our experiments showed any evidence of mammalian Archease enhancing the activity and/or specificity of NSUN2 as a tRNA methyltransferase, neither in intron-containing tRNAs nor in intron-less tRNAs.

It should be also noted that in yeast, no orthologue of Archease is encoded in the genome^10^ whereas two orthologues of Trm4 are found in *Schizosaccharomyces pombe*, named Trm4a and Trm4b^24^. Both proteins were shown to be active tRNA m^5^C methyltransferases modifying different tRNA positions: C34 and C48 for Trm4a, and C49 for Trm4b, respectively^24^. Similarly, plant genomes lack an orthologue of Archease^10^ and encode two Trm4 genes^25^; however, while *A. thaliana* Trm4b was shown to be primarily responsible for tRNA m^5^C methylation, the specificity of Trm4a remains unclarified^26^. Thus, it appears that multiple solutions arose during evolution to enable the members of the NSUN family of tRNA methyltransferases to modify diverse target tRNAs, and different positions within those RNAs. In archaea, the recruitment of the accessory protein Archease improves the target specificity, especially at extremely high temperatures^9^, while duplication and divergence of the modification enzymes in yeast (and possibly plants) enables target selection^24,26^. However, mammals seem to have adapted the single protein NSUN2 to achieve multi-site specificity. During the revision of this manuscript, the cryo-EM structure of human NSUN2 bound to in vitro transcribed human tRNAs was published^27^; the authors show that NSUN2 alone is sufficient to induce extensive structural rearrangement leading to unfolding of the target tRNA and partial melting of the T-stem to allow methylation of C49 and C50, with a preference for methylation of the sequence (m^5^)CNNRR. Part of our experiments employed a mixed system of mouse tRNAs and human recombinant enzymes. While this may represent a limitation of the current study, considering the nearly perfect conservation of tRNAs between the two species^28^, it is unlikely that using human derived tRNAs would yield different outcomes. The lower methylation extent at C48 compared to C49 is consistent with the observed general lower level of m^5^C48 in multiple eukaryotes^7,8,14,29,30^, and possibly represents a preference of NSUN2 for C49 selection. The generally lower methylation extent achieved by NSUN2 on cell-derived tRNA (Fig. 2) compared to the in vitro transcribed tRNA^Asp^ substrate (Fig. 3) may be due to isodecoder composition, modifications present in cellular tRNAs that interfere with NSUN2 methylation, or suboptimal folding of the RNA after extraction and re-solubilization. Still, it is interesting to note that recombinant human NSUN2 was very active in vitro on mitochondrial tRNAs derived from mouse cells, as compared to cytosolic tRNAs (Fig. 2); this may be due to the presence of other modifications that act as determinants or anti-determinants for modification by NSUN2, to higher intrinsic flexibility of mitochondrial tRNAs or to other, yet unknown factors aiding the activity of NSUN2 on cytosolic tRNAs. Our findings demonstrate that investigating tRNA modification in different organisms can still hold surprises, and highlights that diverse strategies have been selected throughout evolution to ensure correct tRNA maturation and function.

## Supplementary Information

Below is the link to the electronic supplementary material.

Supplementary Material 1

Supplementary Material 2

Supplementary Material 3

## Data Availability

All sequencing data were deposited in the Sequence Read Archive (SRA) and are available under the accession number PRJNA1440213.The computational pipeline used for data analysis is available at https://github.com/mwaldl/archease.
