# Supplementary material for: The tRNA methylation activity and specificity of NSUN2 are independent of Archease
Source: Sci Rep. 2026 Jul 29;16:34876. doi: 10.1038/s41598-026-63726-2 (PMC13713551; doi:10.1038/s41598-026-63726-2)
Supplement: Supplementary file 1 [file 41598_2026_63726_MOESM1_ESM.pdf]

Supplementary information

The tRNA methylation activity and specificity of NSUN2 are independent of Archease

Valentina Stuchlik[1], Maria Waldl[1,2,3,4], Anming Huang[5], Moritz Kleinwächter [6,7], Anna Rocchegiani[1], Fatinah El-Isa[1], Ivo L. Hofacker[2,8], Javier Martinez[6], Alexandra Lusser[5] and Elisa Vilardo[1*]

[1] Center for Anatomy & Cell Biology, Medical University of Vienna, Austria

[2] Department of Theoretical Chemistry, University of Vienna, Austria

[3] Vienna Doctoral School in Chemistry (DoSChem), University of Vienna, Austria

[4] Institute of Computer Science and Interdisciplinary Center for Bioinformatics, Leipzig University, Germany

[5] Institute of Molecular Biology, Biocenter, Medical University of Innsbruck, Austria

[6] Max Perutz Labs, Medical University of Vienna, Vienna Biocenter Campus (VBC), Austria.

[7] Vienna Biocenter PhD Program, a Doctoral School of the University of Vienna and the Medical University of Vienna, Austria

[8] Faculty of Computer Science, Research Group Bioinformatics and Computational Biology, University of Vienna, Austria

[*]To whom correspondence should be addressed.

Tel: +43 1 40160 37724; Email: elisa.vilardo@meduniwien.ac.at

Description of Supplementary Files

Supplementary File 1: This document, containing Supplementary methods, figures, table, references.

Supplementary File 2: Summary of tRAM-seq results for Archease KD experiments in comparison to control mESCs. Column identity is indicated in the file. Only positions with minimum coverage of 100 reads and C-retention rate higher than 0.1 in at least one of the samples are listed.

Supplementary File 3: Summary of tRAM-seq analysis for *in vitro* methylation with NSUN2 alone or supplemented with Archease, at 37 °C and at 40 °C. Data were pooled per anticodon family and plotted per position. Only positions minimum coverage of 500 reads and with C-retention rate higher than 0.1 in at least one of the samples are listed.

Supplementary Methods

NGS analyses were performed using the mouse reference genome GRCm39/m39. The reference tRNA set comprised 400 high-confidence mature cytosolic tRNAs predicted by tRNAscan-SE[1] and obtained from the GtRNAdb[2], as well as 22 mitochondrial tRNAs annotated in RefSeq[3] (NC_005089.1; GenBank: AY172335.1). To generate a non-redundant reference, identical mature tRNA sequences (originating from multiple genomic loci) were collapsed, yielding 217 cytosolic and 22 mitochondrial tRNAs.

Bisulfite-treated mESC Archease knockdown (KD) libraries were demultiplexed using bcl2fastq, and adapter-trimmed using cutadapt[4] (-q 10, -e 0.15, -u 3, -a NNNNNNCTGTAGGCACCATCAAT;min_overlap=10). CCA tails (after bisulfite treatment TTA) were removed with cutadapt (-a CCAX=min_overlap=3; -a TTAX=min_overlap=3), and TGIRT-derived 5′ leading Ts were trimmed (-g XTTTTT;min_overlap=1). Reads were further filtered for quality and length using fastp[https://doi.org/10.1093/bioinformatics/bty560] (-A, --length_required=20, --low_complexity_filter, --complexity_threshold=30). Quality control was performed with fastp and custom scripts after each pre-processing step. Preprocessed reads were mapped to the reference using segemehl[5] (v0.3; -i ref.ctidx, -j ref.gaidx, --accuracy 90, -F 1) and mapping rates summarized. C-retention rates were estimated from the resulting mapping using haarz.x (provided by the segemehl v0.3 package; callmethyl). Cytosine positions with a minimum coverage of 100 reads in at least one library were retained for downstream analysis. Modification rates were summarized and plotted per anticodon family; median C-retention throughout each sample were subtracted for background normalization. C-retention rates in the two replicates of KD control and Archease KD were compared with Graphpad Prism by multiple unpaired t test with correction for multiple comparison using the Holm-Sidak method; no cytosine site had p value smaller than 0.05.

Bisulfite-treated *Nsun2* knockout MEF libraries were demultiplexed and adapter-trimmed using cutadapt (-q 10, -e 0.2, -a <adapter>;min_overlap=8). Unique molecular identifiers (UMIs) were extracted using umi_tools[6] (extract, --extract-method=regex --bc-pattern="(?P<umi_1>.{3})([AGCTUN].*)(?P<umi_2>.{6})$"), and TGIRT-derived 5′ leading Ts were trimmed (-g XTTTTT;min_overlap=1). Quality control was performed with fastp and custom scripts after each preprocessing step. Mitochondrial tRNA reference sequences were extended by “CCAN”. Preprocessed reads were mapped using segemehl (v0.3; -i ref.ctidx, --accuracy 90, -maxinterval

200, -difference 2, -evalue 500, -F 1), and mapping rates were summarized. Reads mapping to multiple references with identical scores were assigned randomly. Relative abundances of individual tRNAs were calculated for quality control. For each reference position, aligned nucleotide identities were summarized, and m^5C modification rates were calculated as C-retention fractions (number of reads with C divided by the sum of reads with C and T at that position). In addition, C-retention rates were aggregated at the anticodon family level by combining counts across equivalent positions. Equivalent positions were identified by aligning reference sequences to the Rfam[7] tRNA seed alignment (RF00005) using cmalign[8] (--mapali). m^5C rates for individual tRNAs and anticodon families were filtered for minimum coverage, summarized in TSV files, and visualized.

Supplementary Table 1 Oligonucleotides used in the study.

Name	Sequence (5'-3')
shRNA for *archease* knock-down	
Archease KDsh2_fw	CCGGAAAGACTCGTGAAGAGCTGATCTCGAGATCAGCTCTTCACGAGTCTTTTTTTTG
Archease KDsh2_rev	AATTCAAAAAAAAGACTCGTGAAGAGCTGATCTCGAGATCAGCTCTTCACGAGTCTTT
Reverse transcription qPCR	
qTbp_fw	GGGAGAATCATGGACCAGAA
qTbp_rev	CCGTAAGGCATCATTGGACT
qArchease_fw	ATGGCCATGTTTGGTTACATG
qArchease_rev	CCGGGGTATGAAGTACTCATC
Library preparation: pre-adenylated, barcoded adapters	
i1	p-NNNNNNatcgtCTGTAGGCACCATCAAT-ddC
i2	p-NNNNNNagctaCTGTAGGCACCATCAAT-ddC
i3	p-NNNNNNgcataCTGTAGGCACCATCAAT-ddC
i4	p-NNNNNNtctagCTGTAGGCACCATCAAT-ddC
i5	p-NNNNNNacagtCTGTAGGCACCATCAAT-ddC
i6	p-NNNNNNtagctCTGTAGGCACCATCAAT-ddC
i7	p-NNNNNNactgaCTGTAGGCACCATCAAT-ddC
Cloning primers	

hsaNSUN2_3f	ACTGCATATGGGGCGGCGGTCGC
hsaNSUN2_1r	CATGCTCGAGTCACCGGGGTGGATGGACCC
T7_HH_nAsp(GTC)2_1f	AATTCTAATACGACTCACTATAGGGAGAACGAGGACTGATGAGTCCGTGAGGACGAAACGGTACCCGGTACCGTCTCCTCGTTAGTATAGTGGTGAGTATCCCCGCCTGTCA
T7_HH_nAsp(GTC)2_1r	CGCGTGACAGGCGGGGATACTCACCACTATACTAACGAGGAGACGGTACCGGGTACCGTTTCGTCCTCACGGACTCATCAGTCCTCGTTCTCCCTATAGTGAGTCGTATTAG
BsaI_Asp_AvaII_HDV_1f	CGCGGGAGACGGGTCGGCATGGCATCTCCACCTCCTCGCG
BsaI_Asp_AvaII_HDV_1r	GACCGCGAGGAGGTGGAGATGCCATGCCGACCCGTCTCC
Two-way ligation	
n_Asp(GTC)2_splint2	GGGAATCGAACCCCGGTCTCCCGCGTGACAG
hAsp49-76 (RNA)	CGGGGUUCGAUUCCCCGACGGGGAGCCA

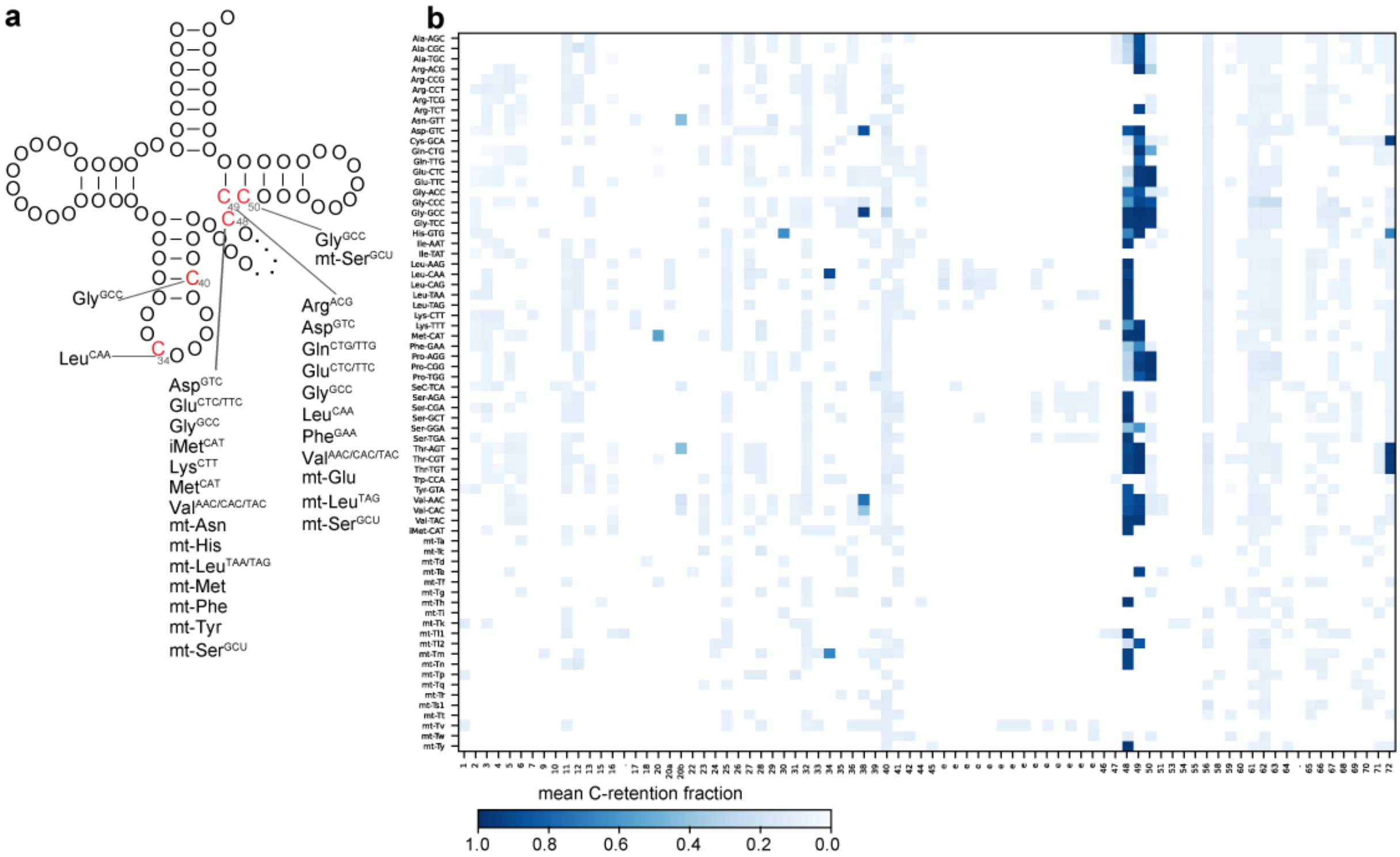

Supplementary Figure S1 m^{5}C methylation landscape of tRNAs. (**a**) Graphical representation of tRNA in the canonical clover-leaf structure. Known target cytosines methylated by NSUN2 are highlighted in red and numbered according to canonical numbering. Nuclear-encoded and mitochondrial tRNAs in which the specific modification site was previously reported in human and/or mouse are listed[6-8,12]; the specific isoacceptor is specified where relevant. (**b**) tRNA m^{5}C methylation landscape of control mESCs according to C-retention rate after bisulfite treatment and tRAM-seq analysis. Positions encoding cytosines are shown in shades of blue according to the C-retention rate (mean of two biological replicates). Positions where no cytosine is encoded are shown in white. The m^{5}C34 in tRNA$^{Leu(CAA)}$, m^{5}C40 in tRNA$^{Gly(GCC)}$, and m^{5}C48-49-50 in cytosolic tRNAs are *bona fide* targets of Nsun2. The m^{5}C38, m^{5}C34 in mt-tRNA^{Met} (mt-Tm), and m^{5}C72, are *bona fide* targets of Dnmt2; Nsun3, and Nsun6, respectively. C retention at positions 20-20b is due to cytosine misincorporation at acp^{3}U sites being mapped to alternative isodecoders encoding cytosine at that position.

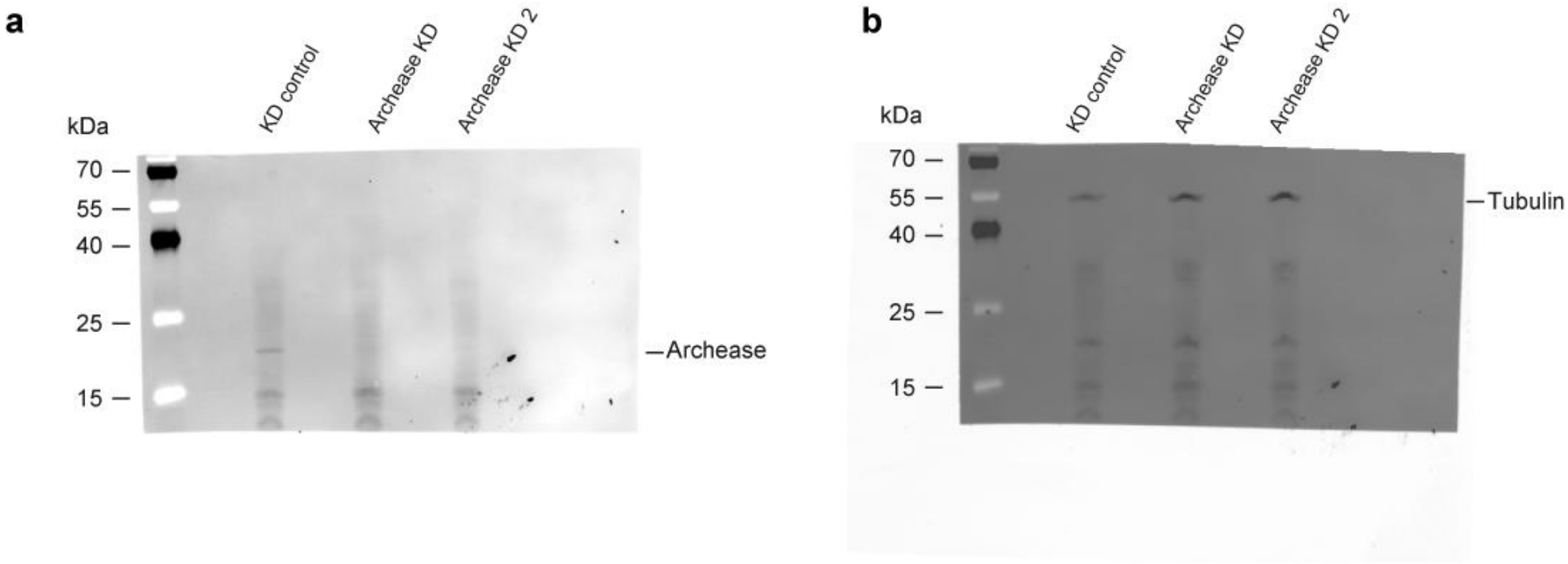

Supplementary Figure S2 Knock down of Archease in mESCs. Source images for cropped panels in Fig. 1a are shown; here the original edges of the membrane are visible. (**a**) Membrane showing the signal after incubation with anti-Archease antibody, and (**b**) after incubation with anti-Tubulin antibody. On the left, the protein standard ProSieve QuadColor Protein Marker (Lonza) was loaded; the molecular weight of selected bands is indicated. On the right, an additional sample of Archease KD mESCs was loaded; this sample was not further used. The stacking and top part of the gel were removed prior to blotting.

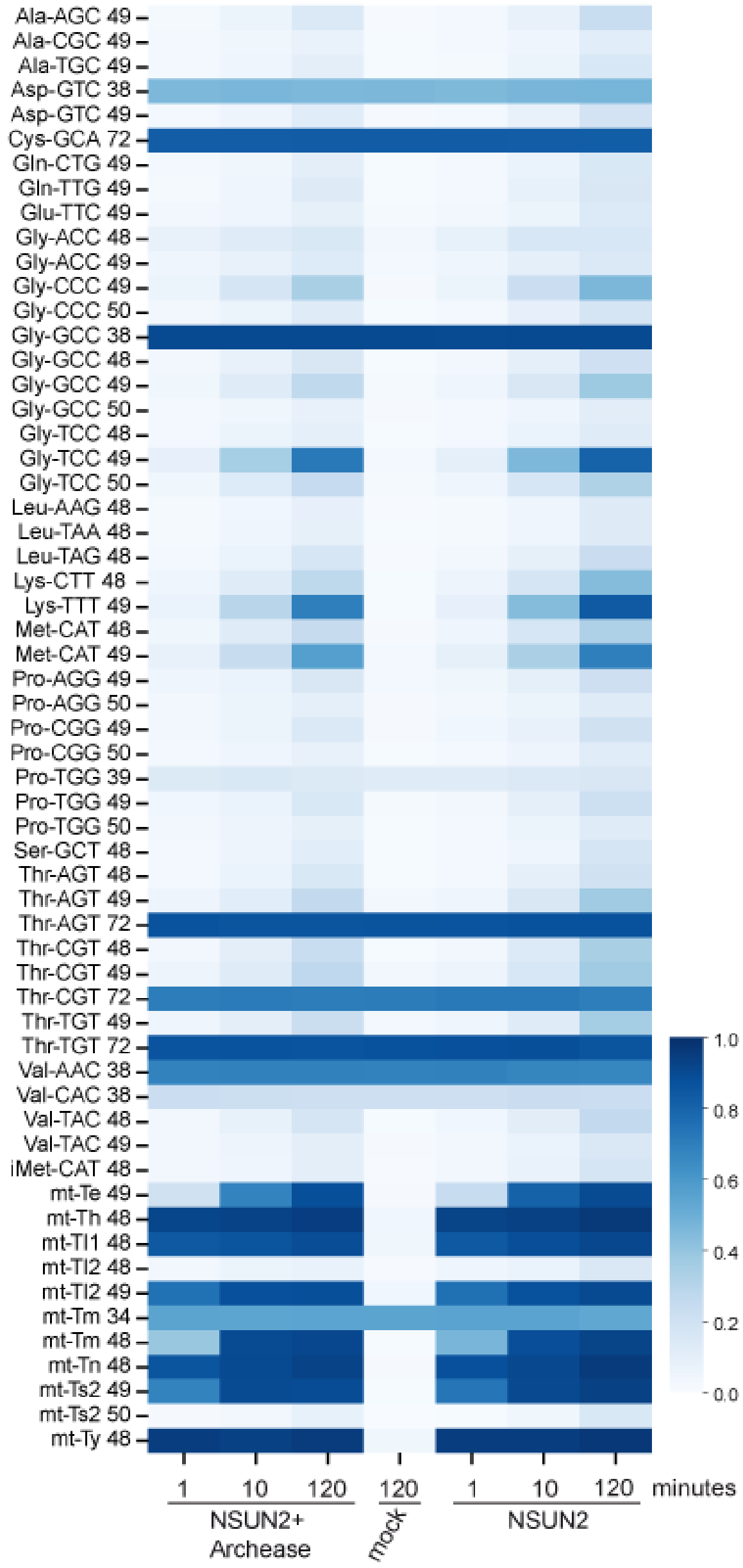

Supplementary Figure S3 Methylation of cellular tRNAs by NSUN2 in presence or absence of Archease at 40 °C. As in Figure 2, RNA was extracted from Nsun2 KO MEFs and incubated at 40 °C with NSUN2 with or without Archease, then processed for tRAM-seq analysis. Quantification of C-retention fraction (thus m^{5}C methylation) throughout the tRNA transcriptome is shown after different incubation times. Data are shown per anticodon family; on the Y axis tRNA anticodon identity and cytosine position are specified. Only positions with minimum coverage of 500 reads, where references encoding a cytosine represent a fraction of at least 0.1 of the total, and with a minimum C-retention fraction of 0.1 are shown. Sites with C-retention signal deriving from other modifications were omitted. Cytosines with high C retention in all samples represent targets of Dnmt2 (C38), Nsun3 (C34), and Nsun6 (C72). Cytosines with C-retention higher in the mock sample than in the treated samples represent *bona fide* background due to incomplete bisulfite conversion.

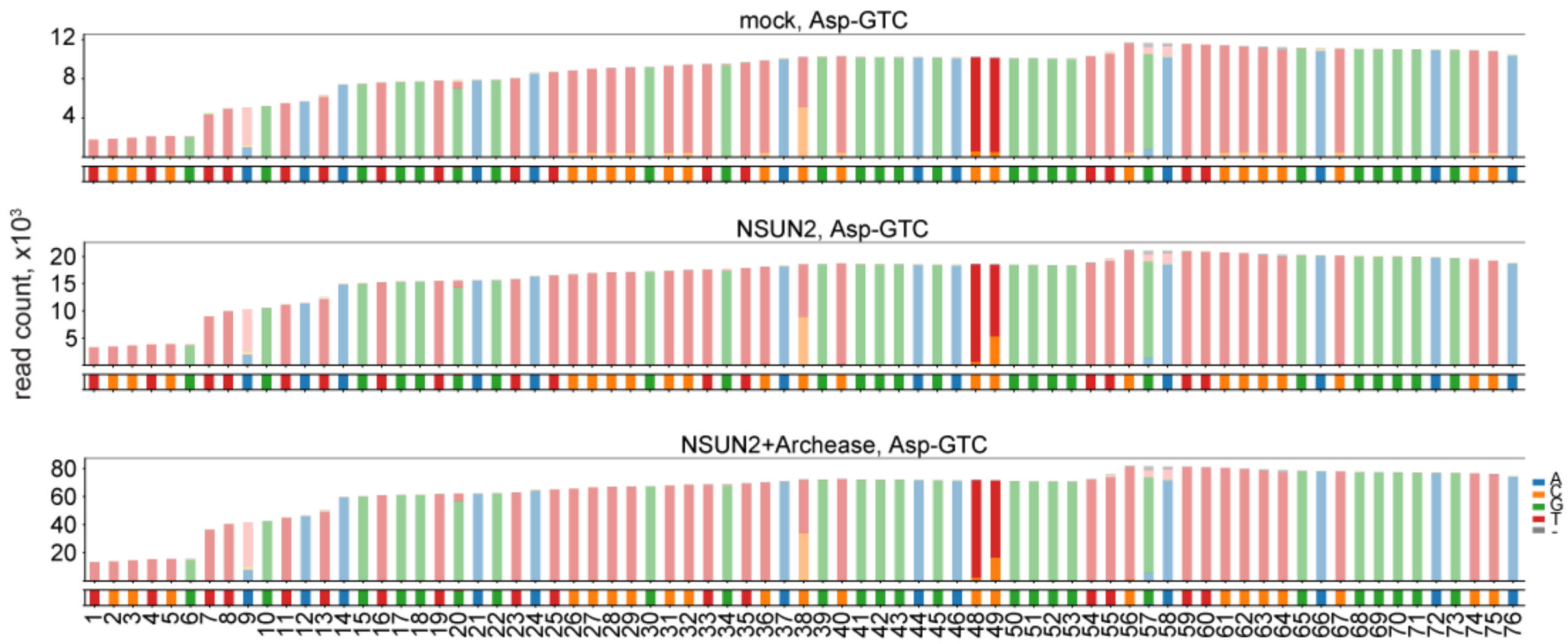

Supplementary Figure S4 Methylation of cellular tRNA^{Asp} by NSUN2 in presence or absence of Archease. Coverage plots with reference nucleotide identity for tRNA^{Asp}, for RNA isolated from NSUN2 KO MEFs and incubated at 37 °C with NSUN2 with or without Archease, or with no enzyme (mock). The different colors indicate the base identity, grey indicates deletion (see legend). Dark colours highlight C48 and C49, which are the expected targets of NSUN2; orange indicates C-retention. The C-retention observed at C38 is due to methylation by DNMT2 in the substrate tRNA; low levels of C-retention throughout the tRNAs is putatively due to marginally incomplete bisulfite conversion. The high extent of misincorporation at A9 is due to m^{1}A modification.

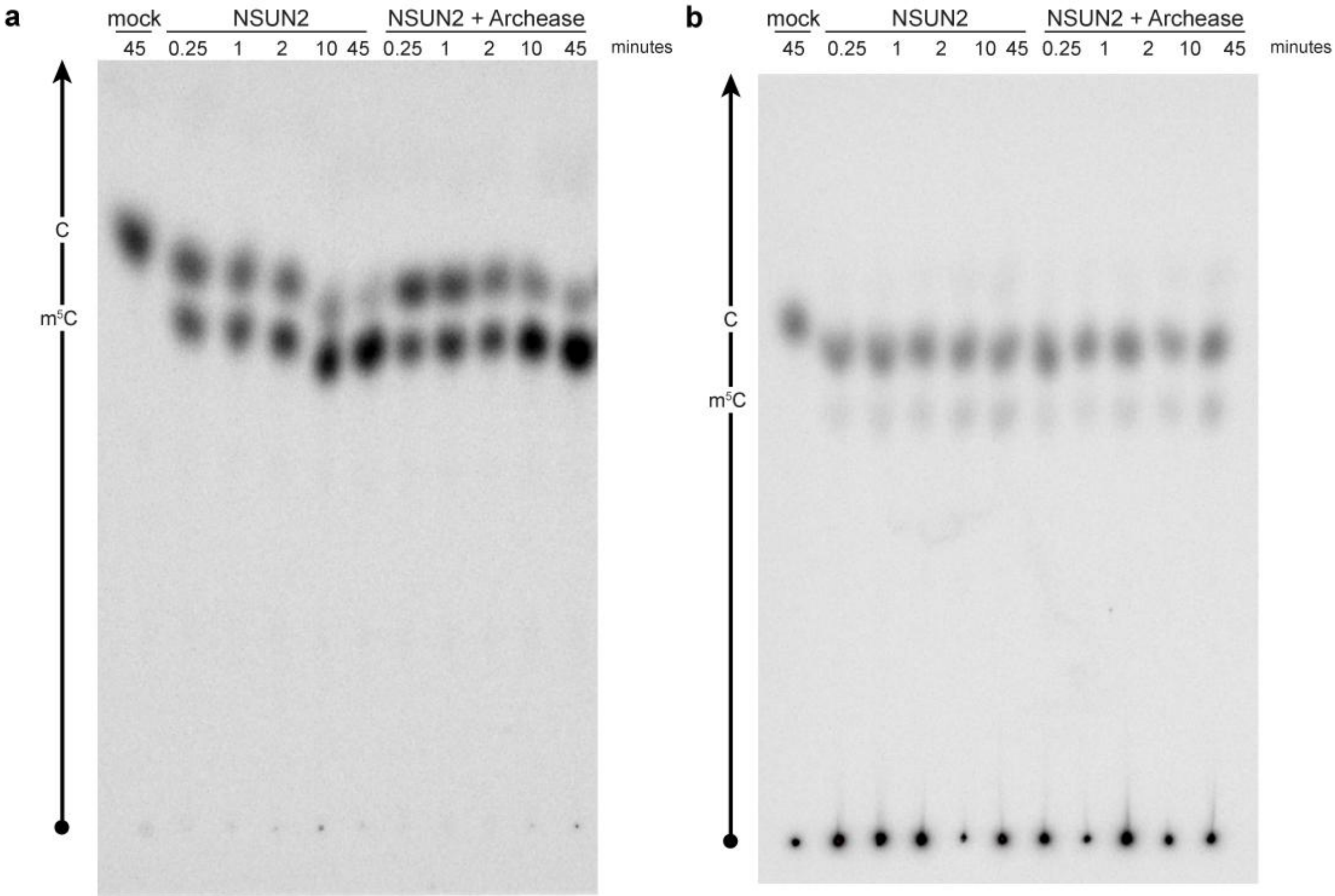

Supplementary Figure S5 ***In vitro* methylation of tRNA^{Asp}.** (**a**) Uncropped TLC from tRNA^{Asp} C49 *in vitro* methylation experiment shown in Figure 3c. The arrow indicates the direction of migration of the solvent, and the identity of the spots is shown on the left. The dot indicates the site of sample application. (**b**) Uncropped TLC from tRNA^{Asp} C48 *in vitro* methylation experiment shown in Figure 3e. The arrow indicates the direction of migration of the solvent, and the identity of the spots is shown on the left. The dot indicates the site of sample application.

SUPPLEMENTARY REFERENCES

1 Chan, P. P., Lin, B. Y., Mak, A. J. & Lowe, T. M. tRNAscan-SE 2.0: improved detection and functional classification of transfer RNA genes. *Nucleic Acids Res* **49**, 9077-9096, doi:10.1093/nar/gkab688 (2021).

2 Chan, P. P. & Lowe, T. M. GtRNAdb 2.0: an expanded database of transfer RNA genes identified in complete and draft genomes. *Nucleic Acids Res* **44**, D184-189, doi:10.1093/nar/gkv1309 (2016).

3 Goldfarb, T. *et al.* NCBI RefSeq: reference sequence standards through 25 years of curation and annotation. *Nucleic Acids Res* **53**, D243-D257, doi:10.1093/nar/gkae1038 (2025).

4 Martin, M. Cutadapt removes adapter sequences from high-throughput sequencing reads. *EMBnet.journal* **17**, 10-12, doi:https://doi.org/10.14806/ej.17.1.200 (2011).

5 Otto, C., Stadler, P. F. & Hoffmann, S. Fast and sensitive mapping of bisulfite-treated sequencing data. *Bioinformatics* **28**, 1698-1704, doi:10.1093/bioinformatics/bts254 (2012).

6 Smith, T., Heger, A. & Sudbery, I. UMI-tools: modeling sequencing errors in Unique Molecular Identifiers to improve quantification accuracy. *Genome Res* **27**, 491-499, doi:10.1101/gr.209601.116 (2017).

7 Kalvari, I. *et al.* Rfam 14: expanded coverage of metagenomic, viral and microRNA families. *Nucleic Acids Res* **49**, D192-D200, doi:10.1093/nar/gkaa1047 (2021).

8 Nawrocki, E. P. & Eddy, S. R. Infernal 1.1: 100-fold faster RNA homology searches. *Bioinformatics* **29**, 2933-2935, doi:10.1093/bioinformatics/btt509 (2013).
